# Origami and Kirigami Structure for Impact Energy Absorption: Its Application to Drone Guards

**DOI:** 10.3390/s23042150

**Published:** 2023-02-14

**Authors:** Chan-Young Park, Yoon-Ah Lee, Jinwoo Jang, Min-Woo Han

**Affiliations:** Department of Mechanical, Robotics and Energy Engineering, Dongguk University, Seoul 04620, Republic of Korea

**Keywords:** origami, kirigami, shock absorption, shape memory alloy, drone, soft robot

## Abstract

As the use of drones grows, so too does the demand for physical protection against drone damage resulting from collisions and falls. In addition, as the flight environment becomes more complicated, a shock absorption system is required, in which the protective structure can be deformed based on the circumstances. Here, we present an origami- and kirigami-based structure that provides protection from various directions. This research adds a deformation capacity to existing fixed-shape guards; by using shape memory alloys, the diameter and height of the protective structure are controlled. We present three protective modes (1: large diameter/low height; 2: small diameter/large height; and 3: lotus shaped) that mitigate drone falls and side collisions. From the result of the drop impact test, mode 2 showed a 78.2% reduction in the maximum impact force at side impact. We incorporated kirigami patterns into the origami structures in order to investigate the aerodynamic effects of the hollow patterns. Airflow experiments yielded a macro understanding of flow-through behaviors on each kirigami pattern. In the wind speed experiment, the change in airflow velocity induced by the penetration of the kirigami pattern was measured, and in the force measurement experiment, the air force applied to the structure was determined.

## 1. Introduction

In the early days, drones were used for military purposes in World War I and the Vietnam War [1]. Currently, private and commercial drones are utilized for medical applications, product delivery, photography, and agricultural purposes [2,3,4,5,6,7]. As the scope of the industry expands, flight stability is improving. Obstacle detection and avoidance are achieved using radar, lidar, vision sensors, and optical flow technologies [8,9,10,11,12,13,14,15,16]. Nevertheless, property damage and injuries caused by drone crashes have increased [17,18,19,20,21]. There are numerous causes of drone crashes. Drones do not respond sufficiently to wind gusts and to complex flight situations. Power supply problems are also in play, as is loss of ground communication.

Some researches seek to continue drone flight after a collision or reduce crash damage. The impact resilience of drones using the drone frame itself and drones that can absorb vertical impacts have been studied [22,23,24,25]. They are technologies that implement shock absorption functions in the forms or materials that make up drones. Flight platforms such as Gymball protect drones from both side-to-side and vertical collisions, and they greatly reduce shock effects [26]. However, larger weights and volumes reduce flight capacity. Most drone cages and propeller guards were designed without considering flight performance and, in addition, are tailored only to particular drones [22,23,24,25,26,27,28,29,30,31,32].

Physical-impact-protection systems that minimize load constraints and are adaptable to drones of varying sizes and shapes are required. Origami engineering may be useful; origami has been widely employed to construct lightweight deformable structures. In addition, it has a potential capability for shock absorption applications. This is possible due to the durability and elasticity of origami, as well as its capacity to be created in a variety of sizes and forms [33,34,35,36,37,38,39].

Origami transforms a flat square sheet of paper into a three-dimensional, sculpture-like form via folding, wherein different architectures can be achieved by the angle of folding. Origami imparts elasticity and durability to thin and light materials; it is useful when designing shock-absorption structures. The magic ball origami could absorb impacts; folded parts are deformed during collisions [40,41,42,43,44].

Considering diverse flight conditions and impact situations, actively deforming the origami structure will improve its effectiveness. Shape memory alloys (SMA) are available for deformable structures; SMAs are materials that return to their original form when heated above their phase transition temperature [45,46,47,48]. In particular, SMA springs are highly useful in deformable structures because they can generate a large deformation in a simple and lightweight form.

Here, we present an advanced drone guard constructed using origami structures comprised of thin-film materials. It utilizes the flexibility and lightness of thin film-based origami to shield a drone from impact. Using SMAs, we created three kinds of deformation modes for the origami structure. These deformation modes were chosen in reference to real-world circumstances. In the drop impact test, we investigated the effectiveness of shock absorption with different modes of deformation. Furthermore, we apply several patterns of kirigami, a technique for creating a cutting pattern, to the origami structure. Then, with kirigami-patterned origami structures, aerodynamic characteristics were investigated, including airflow, wind speed, and aerodynamic load testing.

## 2. Results and Discussion

### 2.1. Conceptual Overview of the Proposed Origami–Kirigami Structures

**Figure 1:**
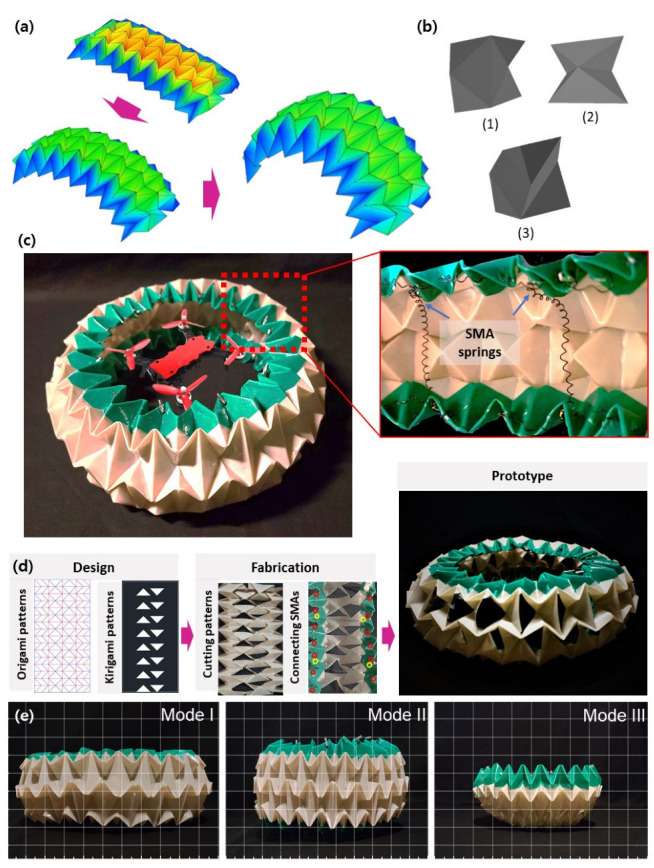
The origami and kirigami constructs. (**a**) Conceptual view of the origami deformation. (**b**) The basic elements of the circular tube. (**c**) The prototype of the impact-absorbing structure incorporating actuators. (**d**) Fabrication process (design the origami and kirigami pattern using computer-aided design software; create the origami and kirigami patterns using automated cutting machine; connect the SMA springs; fold the structure). (**e**) The three variants of the circular tubes.

The origami shown in Figure 1a forms part of the circular tube. The circular tubes can be divided into three elements, shown in Figure 1b. Elements (1) and (2) are joined crosswise; one side serves as a common plane. Element (3) joins elements (1) and (2) upward and downward; there is no common plane. Joined elements (1), (2), and (3) constitute the final structure. As the folding degree of each element can be changed, the circular tube can create a deformation. Based on its three elements, a variety of circular tube structures can be made for different sizes and purposes. The overall tube size can be adjusted by varying the dimensions of basic elements (1), (2), and (3). In addition, the horizontal and vertical ratios can be adjusted. For example, if the vertical ratio is increased, the tube approaches a sphere; if the horizontal ratio is increased, the tube diameter increases.

Figure 1c shows the origami constructs using polyetheretherketone (PEEK) films. PEEK has higher strength and heat resistance than polyethylene terephthalate (PET) and polypropylene (PP) [49,50] (Table 1), so it has been selected as a material for origami structures for drone guards operating through SMA (Table 2) [51,52]. This engineering plastic exhibits excellent heat- and wear-resistance. PEEK can withstand temperatures to 250 °C; in this study, the operating temperature of the SMA actuator ranges from 20 °C to 120 °C. When a current is applied to the SMAs, PEEK serves as an insulator and is preserved during collisions and falls. It is also highly fatigue/impact-resistant with high stiffness; the PEEK-based morphing surface would be useful in repeatable deformation.

An SMA served as an actuator, enabling free switching between the three modes. The SMA springs used in this study are appropriate for circular tubes exhibiting large variations in volume. The SMA spring has a 0.381 mm wire diameter and a 2.54 mm outer diameter. The diameter of the circular tube is inversely proportional to its height, facilitating deformation of the structure. As shown in Figure 1c, three SMA springs were inserted horizontally around the circumference of the circle, and eight were inserted vertically between the top and bottom. Electric wires delivered current to the springs, which caused the joule heating and phase transformation of the SMAs.

Kirigami imparts new characteristics by cutting origami structures into specific patterns or shapes. We drew several kirigami patterns and performed airflow, wind speed, and load cell experiments. Figure 1d shows the circular tube incorporating the kirigami patterns. Also, the fabrication process is shown in Figure 1d.

We focused on three variants of the tube. On the left of Figure 1e, these are modes 1–3. Mode 1 is of large diameter and low height; mode 2 has a small diameter and elevated height; and mode 3 is shaped similar to a lotus flower. Mode 1 is suitable for normal flights; mode 2 protects against collisions and crashes; and mode 3 protects the bottom of the drone. Mode-specific features and volumes are described in detail in the “2.2 Deformation Experiment” section. 

### 2.2. Deformation Experiment

**Figure 2:**
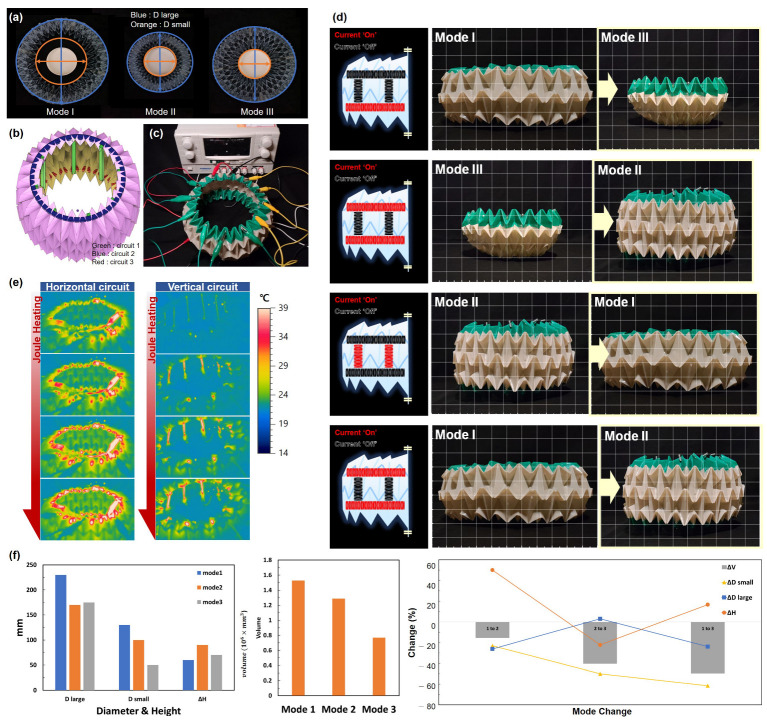
The deformation modes and experiments. (**a**) The three modes of deformation viewed from above. (**b**) Modeling of structure which marks the three SMA circuits. (**c**) Experimental setup for deformation. (**d**) Modes of deformation (Video S1). (**e**) Thermal images when current was applied (Video S2). (**f**) Heights and diameters in the three modes/volume by mode/diameter, height, and volume changes by mode.

SMA actuators were placed around the circumference and in the longitudinal direction of the circular tube to allow free deformation. Stretched SMA contracts one way to the original length when heated. However, as the circumference and height of the circular tube are inversely proportional, SMAs can be used in two-way directionality. If the vertically placed SMAs contract, the circumferential SMAs expand, and vice versa. Figure 2a shows the top views of the three modes. Figure 2b is a model of the circular tube; the areas bearing SMAs are colored green (circuit 1), blue (circuit 2), and red (circuit 3). The SMAs of each color are connected in series and, as shown in Figure 2c, connected to a power supply. In the experimental setup shown in Figure 2c, the structure was transformed by applying current to the SMA springs. As a result of trial and error to determine the proper reaction speed of the SMAs by current, 700 mA was applied to the system.

Figure 2d shows the modes of deformation. When the structure is in mode 1, a current applied to circuit 3 transforms into mode 3. When the structure is in mode 3, a current applied to circuit 2 transforms it into mode 2. When the structure is in mode 2, a current applied to circuit 1 transforms it into mode 1. Finally, a return to mode 2 is achieved by applying current to circuits 2 and 3. These steps can be varied. The deformation sequence is shown in Video S1. The thermal images by Joule heating are observed in Figure 2e and Video S2, following the SMA circuits embedded in the origami structures. 

Figure 2f shows the results of the deformation experiment. In terms of diameter, the mode 1 D_large_ is 230 mm, 35.3% greater than that of mode 2 (170 mm). Mode 1 (with the largest diameter) recognized an impact in a larger range in a horizontal direction. Rapid recognition of an impact risk allows the inertial devices to hold the drone flight level. The mode-2 diameter is 26% less than that of mode 1; mode 2 facilitates drone passage through a narrow area. 

The height of mode 2 (90 mm) is about 50% greater than that of mode 1 (60 mm), and the wide range of the drone was protected against falls. The tube blocked drone debris so it could prevent secondary damage. In other words, mode 2 might have had an effect on protecting both drones and their surroundings against crashes and collisions.

Modes 1–3 have volumes of 1.527 × 10^6^ mm^3^, 1.288 × 10^6^ mm^3^, and 0.12 × 10^6^ mm^3^. Upon transformation from mode 1 to 2, 2 to 3, and 1 to 3, the volume reductions are 15.6%, 92.3%, and 90.8%, respectively. The mode 3 D_small_ is 50 mm, thus 61.5% and 50% less than those of mode 1 (130 mm) and mode 2 (100 mm). The mode 3 D_large_ is 175 mm, thus 23.9% less than that of mode 1 (230 mm) and 2.9% larger than that of mode 2 (170 mm). In other words, mode 3 shows the smallest bottom diameter and the top diameter, which has an intermediate value between mode 1 and mode 2. In mode 3, the protective layer is wrapped around the underside and sides of the drone. Therefore, the lower region of the drone is very much protected. This is valuable for transport drones because their center of gravity is lowered by the cargo, so transport drones fall vertically downward after a crash. 

In summary, mode 1 is suitable for normal flight; mode 2 protects against horizontal impacts and crashes; and mode 3 protects the lower part of the drone. With these morphing guards, the protection is effective because the structure can be deformed to accommodate various situations.

### 2.3. Drop Impact Test

Circular tubes are elastic; they can deform and absorb impacts [53,54]. We performed drop experiments to demonstrate the effectiveness of shock absorption. After deforming the shape, the deformed shape was maintained without additional current supply. Before the impact test, the structure was reshaped into the form of the subject being evaluated. We used a load cell to determine the impact time, force, and extent of the crushing. Acrylic plates that support falling objects were placed on top of the load cell. Horizontal bars marked with reference points were installed to accurately identify drop heights and object locations. We then dropped three types: a PEEK tube, a PEEK tube to which a mass had been attached, and a drone. All data were recorded by high-speed cameras so that we could analyze deformation on impact. A Robotous load cell was used (*RFT40-SA01*). The *F_z_* load capacity was 150 N and the resolution was 200 mN.

**Figure 3:**
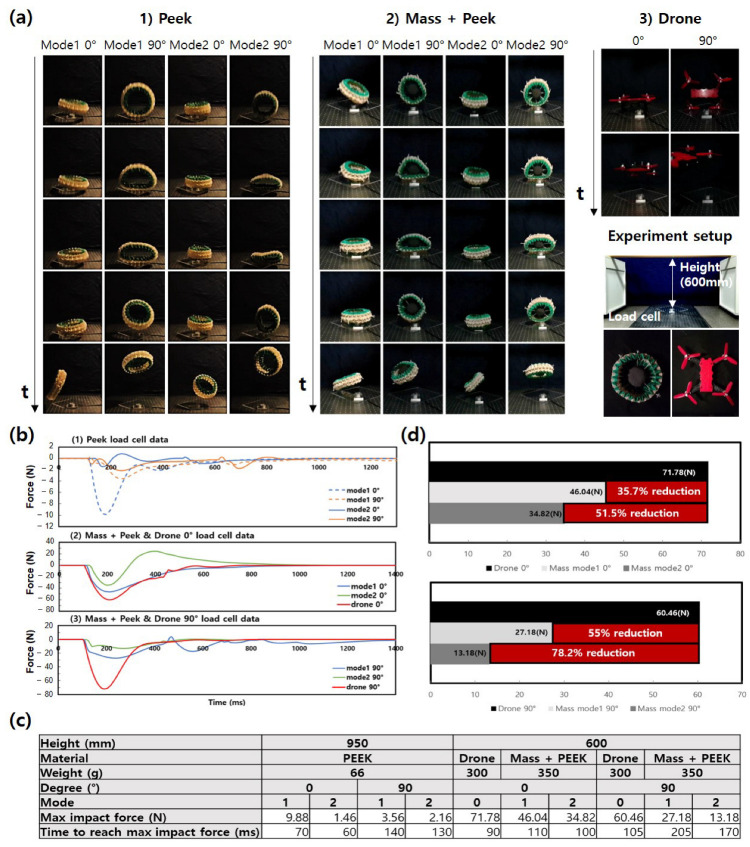
Drop impact test results. (**a**) Photographs of deformations on impact taken by a high-speed camera (Videos S3–S6), setup of the drop impact test. (**b**) Time–impact-force graphs. (**c**) Table with heights, materials, weights, angles, modes, maximum impact forces, and times to the maximum impact forces. (**d**) Maximum impact reduction.

We first dropped the tube made of PEEK to identify deformation on impact. The 60 g structure was dropped from a height of 950 mm. Four experimental conditions can be determined depending on the angle of collision and mode of deformation. In this research, there were two types of collision angles: 0° (frontal) and 90° (side), and two deformation modes (1 and 2, with large diameter/low height and small diameter/high height, respectively) (Figure 3).

Figure 3a-1 shows PEEK deformation, which was both very large and variable over time (Video S3). In particular, the structure bounced after collision, confirming its high elasticity and resilience. The maximum impact forces are shown in Figure 3b,c, which compares mode 1 and mode 2. In the case of 0°, mode 1 exhibited a larger impact force (mode 1, 9.88 N after a fall of 70 ms; mode 2, 1.46 N after a fall of 60 ms). In the case of the 90° comparison, mode 1 showed a larger impact force (mode 1, 3.56 N after a fall of 140 ms; mode 2, 2.16 N after a fall of 130 ms). In terms of the maximum impact force, mode 1 exhibited large values at both 0° and 90°.

Some of the time–impact-force graphs in Figure 3b indicate positive impact forces for two possible reasons. First, after the object hit the acrylic plate, residual plate vibrations may have been measured as a positive impact force. Second, when a structure fell on the edge of the plate, the edge force was affected; this was associated with an upward force in the middle of the plate, recorded as a positive impact force.

Next, we added a mass to the tube to verify that shock absorption was effective even when a drone was connected (Figure 3a-2). As the addition of mass is likely to change the deformation mechanism, we checked the shape changes and maximum impact forces. We attached a 290 g mass equal to the medium-small drone (Mjx R/C, Bugs 8 Pro) to the PEEK structure. The weight of the PEEK structure was 60.7 g. After attaching the 290 g mass, the total mass was 350.7 g. The 290 g mass was produced using two 100 g pendulums, one 50 g pendulum, and polymer clay. After passing four rubber bands through the PEEK structure, the mass was tied to the bands. Considering the load cell capacity (150 N), the drop height was reduced to 600 mm. Figure 3a-2 shows the time-dependent behavior of the mass with a PEEK structure (Video S4). The bouncing was smaller than that of the PEEK tube alone, but it could return to its original shape after the collisions. The result showing that the elasticity and resilience were sufficiently high was obtained through bounce after the collision. 

We then measured the maximum impact forces directly related to the destruction of the drones. Figure 3b-2 shows the mass with a PEEK structure and a drone at a collision angle of 0°. The drone exhibited a maximum impact force of 71.78 N at 90 ms after touching the plate. Mode 1 of mass with PEEK collision exhibited a maximum impact force of 46.04 N at 110 ms after reaching the plate, and the mode 2 collision had a maximum impact force of 34.82 N at 100 ms after the drop. 

Figure 3b-3 shows the mass with a PEEK structure and a drone at a collision angle of 90°. The drone exhibited a maximum impact of 60.46 N at 105 ms. In mode 1, the mass with a PEEK collision exhibited a maximum impact force of 27.18 N at 205 ms after the drop; the mode 2 value was 13.18 N 170 ms after the drop. The maximum impact force gradient was severe regardless of the angle of falling for the drone after impacts; however, the origami guards had a longer collision duration.

Figure 3d shows the reduction of the maximum impact force of the mass with PEEK compared to the drone. In a head-on collision (0°), the maximum impact force of mode 2 was 51.5% less than that of the drone, and 35.7% less for mode 1. In a side collision (90°), the maximum impact force of mode 2 was 78.2% less than that of the drone, and 55% less for mode 1. 

In addition, drop experiments were conducted with the drone (Figure 3a-3 and Video S5) and thin films (Polyvinyl chloride, PVC), the results of which are attached as appendices (Video S6). In the future, we would like to integrate comparison testing data with commercial drone guards and investigate shock absorption effects while considering the design problems of the connection architecture between the drone body and the guard.

**Figure 4:**
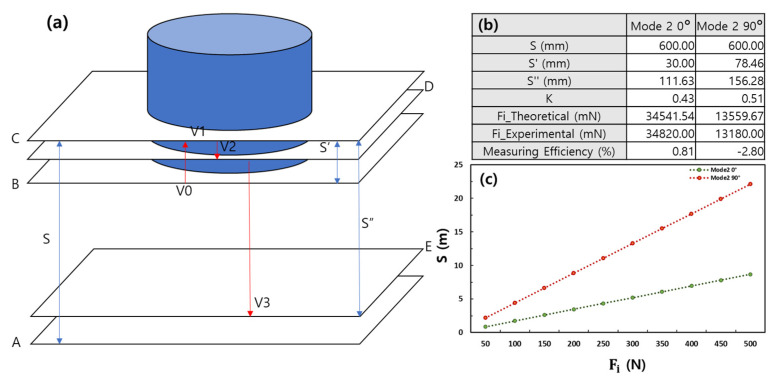
(**a**) Mathematical modeling of shock absorption on impact. The chronological positions of the acrylic plates are denoted by A–E. The structure is the blue cylinder. The structure itself falls, but we assume in this model that the acrylic plate moves up to the structure. *V*_0_: velocity at which the structure first hits the acrylic plate, *V*_1_: velocity at which the structure is maximally crushed and completely stops after hitting the plate, *V*_2_: velocity at which the structure falls off the acrylic plate after completely stopping on the plate, *V*_3_: velocity at which the structure bounces up and completely stops in the air, *S*: height from which the structure is dropped, *S*′: maximum extent of structure crushing, and *S*″: maximum height from which the structure bounces after hitting the plate. (**b**) Theoretical and experimental impact forces. (**c**) Theoretical impact force *F_i_*–*S* graph/*S*, *S*′, *S*″, and *K* are equal to the value at (**b**).

A theoretical model for shock absorption structures is shown in Figure 4a. This model is designed with the assumption that it is completely reversible. As the time and distance between *V*_0_ and *V*_2_ are very small, we assumed that the accelerations were equivalent [55,56,57]. Acceleration from *V*_0_ to *V*_2_ is termed *a*. Thus, $2aS\prime =V_{1}^{2}-V_{0}^{2}$ because it is also assumed that the accelerations are identical from *V*_0_ to *V*_1_. As *V*_1_ = 0, $V_{0}=\sqrt{2gS}$ and $a=-gS/S^{\prime }$. The time

$$t^{\prime }=\frac{S^{\prime }(2+\sqrt{2)}}{\sqrt{gS}}$$

is that from *V*_0_ to *V*_1_ with acceleration *a*. *K* is the coefficient of repulsion. As $KV_{0}=V_{2}=at\prime \prime$, the time from *V*_1_ to *V*_2_ is

$$t^{\prime \prime }=\frac{K\sqrt{2gS}}{gS/S^{\prime }}\;$$

The delta time from *V*_0_ to *V*_2_ is

$$\Delta t=t^{\prime }+t^{\prime \prime }=\frac{\sqrt{2}S\prime }{\sqrt{gS}}+\frac{K\sqrt{2gS}}{gS/S^{\prime }}$$

The momentum change from *V*_0_ to *V*_2_ is $\Delta P=m\sqrt{2gS}\left(K+1\right)$ because when the velocity is calculated as a vector, $\Delta P=mV_{2}-mV_{0}=mV_{0}\left(K+1\right)$. The impact force from *V*_0_ to *V*_2_ is

$$F_{i}=\frac{\Delta P}{\Delta t}=\frac{m\sqrt{2gs}\left(1+K\right)}{S\prime \left(2+\sqrt{2}\right)/\sqrt{gS}+K\sqrt{2gS}/\left(gS/S\prime \right)}=\frac{mgS\sqrt{2}\left(1+K\right)}{S\prime \left(2+\sqrt{2}\left(1+K\right)\right)}$$

As, $V_{2}=\sqrt{2gS\prime \prime }$, $V_{0}=\sqrt{2gS}$

$$K=\frac{V_{2}}{V_{0}}=\frac{\sqrt{2gS^{\prime \prime }}}{\sqrt{2gS}}=\sqrt{\frac{S^{\prime \prime }}{S}}$$

*K* can be obtained by measuring the height (*S*″) of the structure that bounces after collision. *S*′ can be obtained by measuring the maximum extent of crushing after the structure collides with the acrylic plate. The theoretical impact force is obtained by the insertion of these values into the formula

$$F_{i}=\frac{mgS\sqrt{2}\left(1+K\right)}{S\prime \left(2+\sqrt{2}\left(1+K\right)\right)}$$

Experimental accuracy is determined by comparing this theoretical value to the maximum impact force.

Figure 4b shows that in mode 2 at 0° the theoretical impact force is 34.54 N; the experimental value was 34.82 N (0.8% error). In mode 2 at 90°, the theoretical impact force is 13.56 N and the experimental force was 13.18 N (−2.8% error). The small errors confirm the accuracy of the real-world experiments. 

Figure 4c shows the *F_i_-S* graph. It is assumed that *S*′ is always maximum crushing when dropped from the *S* range of the graph. The expression *F_i_* can be used to optimize the design by varying the *S*′ and *k*′ values of the circular tube. This is possible because our shock-absorbing structures are largely unrestricted by material and size. The maximum upper-limit height can be obtained by adding factors such as *S*′ and *K* to the *F_i_–S* graph.

### 2.4. Airflow Testing

A shock-absorption structure was mounted around the drone, thus affecting airflow. To find flight-friendly protection, we added a kirigami pattern to the origami structure, allowing the air to pass through the kirigami perforations. The portions of the circular tube were constructed from polyvinyl chloride, which is very transparent and folds smoothly (297 mm wide and 148.9 mm long). Then we grafted four kirigami patterns. The kirigami patterns were designed using CAD-based software and created using an automatic cutter. We observed the airflow, wind speed, and aerodynamic forces of each pattern. In all experiments, the airflow from the outside to the inside of the deformable guards (outside) and the airflow from the inside to the outside (inside) were measured, respectively.

Model 1 served as the control group (no pattern). Models 2 and 3 feature triangular holes with different directions. Models 4 and 5 feature circular kirigami patterns with different areas. Models 2, 3, and 5 have kirigami pattern areas of 5535 mm^2^; the model 4 area is 2650.8 mm^2^. Model 4 limits the area of the kirigami pattern by 52% compared to other models.

**Figure 5:**
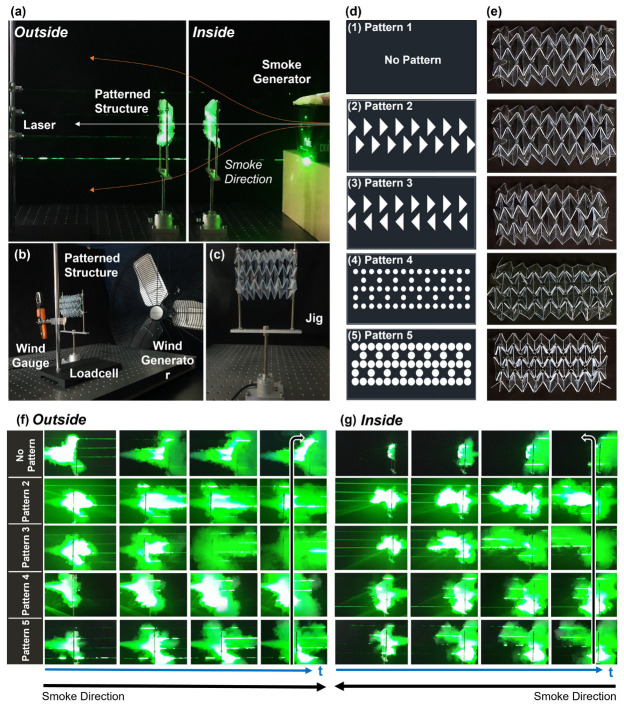
Air flow testing. (**a**) The flow experiment showing the laser, the smoke generator, the jig, and the origami structures with kirigami patterns. We use the terms “outside” when smoke is sprayed from the outside onto a structure, and “inside” when smoke is sprayed from the inside onto a structure. (White arrow—passing through a structure, orange arrows—spreading.) (**b**) The wind speed and load cell experimental setup. The load cell and wind speed experiments were performed in a single session. (**c**) The jig that fixes the structure to the load cell. (**d**) The kirigami patterns. (**e**) Origami structures with kirigami patterns. Flow test results; (**f**) Outer flows over time (Video S7). (**g**) Inner flows over time (Video S8).

We first performed smoke experiments to see the macroscopic airflow. The experimental devices were arranged with wind generator, laser, smoke generator, wind gage, loadcell, and deformable guards, as shown in Figure 5. The degree of spread of the smoke over time was qualitatively determined using the laser as a reference line.

In the case of model 1 (the control), the flow tended not to pass through the structure, but rather spread as indicated by the orange arrows in Figure 5a. 

As a result, the larger the size of the kirigami hole, the greater the amount of initial smoke passing through the structure. In the case of model 3, in which the triangular pattern was arranged in the reverse direction, it was possible to confirm the phenomenon of smoke spreading over time. Figure 4f,g shows the flow of smoke that changed with time for each kirigami pattern. A related video is in the Supplementary Materials (Videos S7 and S8).

We performed wind speed and load cell experiments on models 2, 3, and 5, which have the same hole sizes as kirigami patterns. The value of the wind speed and forces were measured contemporaneously.

**Figure 6:**
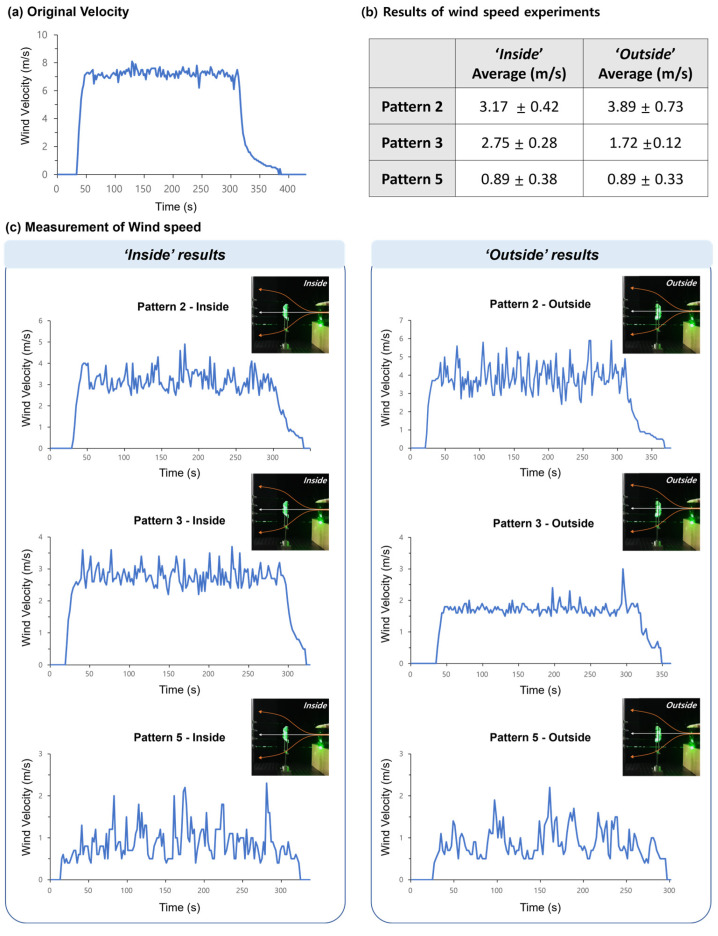
The results of the wind speed experiments. (**a**) Airflow velocity of the generated wind. (**b**) Mean and standard deviation of air speed through each model. (**c**) Wind speed graphs obtained when the wind passed the origami structures.

In wind speed experiments, air velocity is measured when flowing through after “outside” and “inside” structures. (Figure 6, Videos S7 and S8). The experiment is set up similarly to the smoke experiment. A wind speed of 8 m/s was generated using the wind generator. As a result, model 2 exhibited the fastest airflow in both cases of ‘inside’ and ‘outside’, with an average of 3.17 m/s and 3.89 m/s, respectively. In the case of model 3, the difference between the maximum and minimum peak values of the wind speed was smaller than that of other kirigami patterns, so the standard deviation value was calculated to be the lowest.

**Figure 7:**
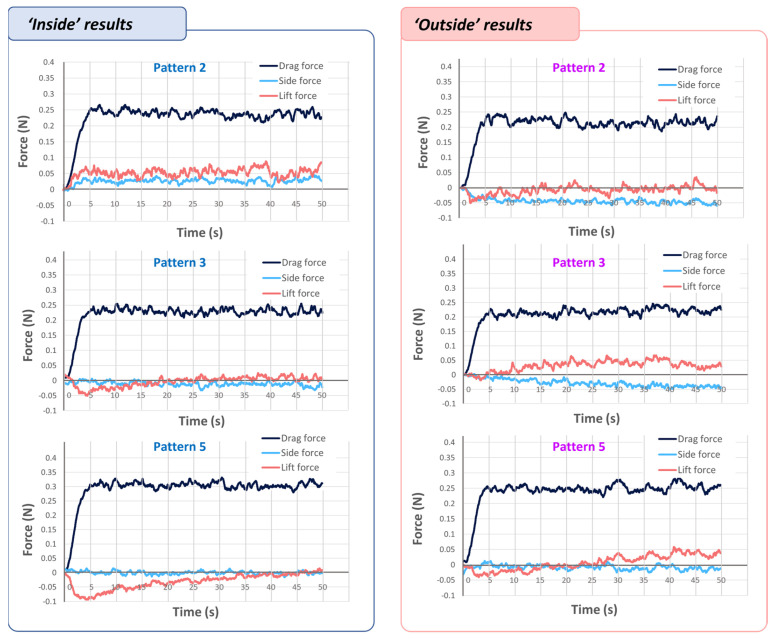
Measurements of the aerodynamic forces with different kirigami-patterned origami structures.

Next, we calculated aerodynamic forces on drag, side, and lift. A load cell was installed at the bottom of the fixing jig where the origami structure is attached. Figure 7 shows the results of the aerodynamic loads with the ‘inside’ and ‘outside’ cases.

In the ‘inside’ case, model 5 had an average value of drag force of approximately 0.3 N, while Models 2 and 3 had a value of approximately 0.25 N, representing an average force value approximately 80% lower than model 5. In the ‘outside’ case, an average drag value of approximately 0.25 N was measured in model 5, and in models 2 and 3, approximately 0.23 N—approximately 90% of the measured value of model 5. The lift and side values were measured to be extremely low in comparison to the drag force.

## 3. Conclusions

Using the origami method, we created a deformable shock-absorbing structure, which can be transformed as needed. The proposed protection system exploits the flexibility and lightness of origami, and it can be applicable to objects of various shapes and sizes. By adding SMAs to a circular tube, we created three variants (Modes 1, 2, and 3). The deformation modes can be selected based on the situation (flight, collision and fall) and the need for bottom-side protection. These modes are implemented through a phase change of each SMA spring, which allows for repeated motions generated through the current. The elastic behavior of PEEK, used as the main material of the origami structure, is combined with the characteristics of the origami pattern to enable a reversible operation. Regarding the viscoelastic properties of PEEK, it has been demonstrated that the temperature of PEEK during SMA operations is lower than the melting point of PEEK. As a result, regardless of the SMA, PEEK exhibits elastic behavior. 

Mode 1’s low height facilitates the usual flight mode. Mode 2’s covering form protects drones in enlarged areas in case of a fall. In addition, after a crash, secondary damage caused by debris can be prevented. Also, the small diameter facilitates passage through narrow areas. Mode 3’s lotus flower form covers the underside of the drone, usefully protecting devices such as cameras and sensors at the bottom of the drone.

Depending on the impact conditions, the origami structure’s mode can be changed to increase the impact time and decrease the impact force. In impact experiments, 350 g of mass with a PEEK structure shows a maximum impact reduction of 35.7% at 0°/mode 1, 51.5% at 0°/mode 2, 55% at 90°/mode 1, 78.2% at 90°/mode 2. Thus, the mode-2 shock absorption was superior to that of mode 1 at any angle; however, both reduced the maximum impact force effectively. 

We added a kirigami pattern to the origami shock-absorption structure to observe flight characteristics, including observation of airflow, wind speed, and aerodynamic forces. Experiments were carried out on four different types of kirigami patterns. The kirigami pattern was divided into two types: those composed entirely of triangle patterns and those composed entirely of circle patterns. The experiment was carried out on two types of triangular pattern specimens, one with the triangles aligned in one direction and the other with the triangles’ directions facing one another. In the case of the circular patterns, an experiment was conducted in which the empty space was the same as the triangular pattern, and the other had approximately half of the empty space.

In the airflow experiment, the larger the size of the kirigami pattern, the smoother the initial air penetration. In the specimens with the triangular kirigami patterns facing one another, the air scattering tendency increased over time. In addition, the fastest wind speed was observed through the specimen with the triangular kirigami pattern aligned in one direction during the wind velocity experiment. In the experiment measuring aerodynamic force, the drag force of the origami structure to which the circular kirigami patterns were applied was the greatest.

This research investigates the shock-absorbing and aerodynamic properties of deformable origami structures incorporating kirigami patterns. More advanced protective structures could be created based on an understanding of soft deployable structures with origami patterns if more research is processed on the design optimization for shock absorption and aerodynamics. Also, the effect of shock absorption is expected to vary based on the kirigami designs. Certain experiment results will be included in future shock absorption studies to provide more thorough experimental observations and model predictions.

## Figures and Tables

**Table 1:** Mechanical and electrical properties of PEEK, PET, and PP [49,50].

	PEEK	PET	PP
Density (kg/m^3^)	1320	1380	900
Tensile strength (MPa)	110	-	27
Tensile modulus (GPa)	4.482	1.2	1.2
Flexural modulus (GPa)	4.14	2.3	1.15
Flexural strength (MPa)	179	67	33
Melting temperature (°C)	334	231	-

**Table 2:** Mechanical and physical properties of NiTi SMA [51,52].

Property of NiTi	Martensite	Austenite
Density (g/cm^3^)	~6.45	
Poisson’s ratio	~0.33	
Ultimate tensile strength (MPa)	Up to 1900	
Young’s modulus (GPa)	25–40	60–83
Yield strength (MPa)	70–140	195–690
Thermal conductivity (W/(m·K))	8.6	18
Coefficient of thermal expansion (K^−1^)	6.6	11
Electric resistivity (Ω·cm)	76	82
Phase transition temperature (°C)	Start (M_s_): 52	Start (A_s_): 68
	Finish (M_f_): 42	Finish (A_f_): 78

## Supplementary Materials

The following supporting information can be downloaded at: https://www.mdpi.com/article/10.3390/s23042150/s1, Video S1: Deformation of the origami structures, comprising of shape memory alloys; Video S2: Thermal response to the applying current; Video S3: Impact testing of the PEEK structure; Video S4: Impact testing of the PEEK structure with mass; Video S5: Impact testing of drone; Video S6: Impact testing of the PVC structure; Video S7: Outer flows over time with different kirigami patterns; smoke is sprayed from the outside onto the origami structure; Video S8: Inner flows over time with different kirigami patterns; smoke is sprayed from the inside onto the origami structure.
